# Aerosolized Hydrogen Peroxide Decontamination of N95 Respirators, with Fit-Testing and Viral Inactivation, Demonstrates Feasibility for Reuse during the COVID-19 Pandemic

**DOI:** 10.1128/msphere.00303-22

**Published:** 2022-08-30

**Authors:** T. Hans Derr, Melissa A. James, Chad V. Kuny, Devanshi R. Patel, Prem P. Kandel, Cassandra Field, Matthew D. Beckman, Kevin L. Hockett, Mark A. Bates, Troy C. Sutton, Moriah L. Szpara

**Affiliations:** a Environmental Health and Safety, Pennsylvania State University, University Park, Pennsylvania, USA; b Animal Resource Program, Pennsylvania State University, University Park, Pennsylvania, USA; c Department of Biology, Pennsylvania State University, University Park, Pennsylvania, USA; d Department of Biochemistry and Molecular Biology, Pennsylvania State University, University Park, Pennsylvania, USA; e Department of Plant Pathology and Environmental Microbiology, Pennsylvania State University, University Park, Pennsylvania, USA; f Department of Veterinary and Biomedical Sciences, Pennsylvania State University, University Park, Pennsylvania, USA; g Department of Statistics, Pennsylvania State University, University Park, Pennsylvania, USA; h Center for Infectious Disease Dynamics, Pennsylvania State University, University Park, Pennsylvania, USA; i Huck Institutes of the Life Sciences, Pennsylvania State University, University Park, Pennsylvania, USA; j Occupational Medicine Program, Pennsylvania State University, University Park, Pennsylvania, USA; Mount Sinai School of Medicine

**Keywords:** N95 respirators, filtering facepiece (FFP) respirators (FFR), decontamination, aerosolized hydrogen peroxide, COVID-19, SARS-CoV2, virologic testing, virus, fit-testing, disinfection, sterilization, CURIS

## Abstract

In response to the demand for N95 respirators by health care workers during the COVID-19 pandemic, we evaluated decontamination of N95 respirators using an aerosolized hydrogen peroxide (aHP) system. This system is designed to dispense a consistent atomized spray of aerosolized, 7% hydrogen peroxide (H_2_O_2_) solution over a treatment cycle. Multiple N95 respirator models were subjected to 10 or more cycles of respirator decontamination, with a select number periodically assessed for qualitative and quantitative fit testing. In parallel, we assessed the ability of aHP treatment to inactivate multiple viruses absorbed onto respirators, including phi6 bacteriophage, herpes simplex virus 1 (HSV-1), coxsackievirus B3 (CVB3), and severe acute respiratory syndrome coronavirus 2 (SARS-CoV-2). For pathogens transmitted via respiratory droplets and aerosols, it is critical to address respirator safety for reuse. This study provided experimental validation of an aHP treatment process that decontaminates the respirators while maintaining N95 function. External National Institute for Occupational Safety & Health (NIOSH) certification verified respirator structural integrity and filtration efficiency after 10 rounds of aHP treatment. Virus inactivation by aHP was comparable to the decontamination of commercial spore-based biological indicators. These data demonstrate that the aHP process is effective, with successful fit-testing of respirators after multiple aHP cycles, effective decontamination of multiple virus species, including SARS-CoV-2, successful decontamination of bacterial spores, and filtration efficiency maintained at or greater than 95%. While this study did not include extended or clinical use of N95 respirators between aHP cycles, these data provide proof of concept for aHP decontamination of N95 respirators before reuse in a crisis-capacity scenario.

**IMPORTANCE** The COVID-19 pandemic led to unprecedented pressure on health care and research facilities to provide personal protective equipment. The respiratory nature of the SARS-CoV2 pathogen makes respirator facepieces a critical protective measure to limit inhalation of this virus. While respirator facepieces were designed for single use and disposal, the pandemic increased overall demand for N95 respirators, and corresponding manufacturing and supply chain limitations necessitated the safe reuse of respirators when necessary. In this study, we repurposed an aerosolized hydrogen peroxide (aHP) system that is regularly utilized to decontaminate materials in a biosafety level 3 (BSL3) facility, to develop a method for decontamination of N95 respirators. Results from viral inactivation, biological indicators, respirator fit testing, and filtration efficiency testing all indicated that the process was effective at rendering N95 respirators safe for reuse. This proof-of-concept study establishes baseline data for future testing of aHP in crisis-capacity respirator-reuse scenarios.

## INTRODUCTION

The early phase of the severe acute respiratory syndrome coronavirus 2 (SARS-CoV-2) pandemic resulted in a shortage of personal protective equipment (PPE). In health care settings, the need for PPE is critical to protect frontline health care providers from infection and to reduce cross-contamination between patients with coronavirus disease 2019 (COVID-19) and other uninfected patients. In health care settings, N95 filtering facepiece (FFP) respirators, including surgical N95 respirators, are used to provide protection from airborne infectious particles. The N95 terminology refers to the ability to block at least 95% of the most penetrating particle sizes (0.1 to 0.3 μm). Proper use of N95 respirators requires qualitative fit-testing (QLFT) or quantitative fit-testing (QNFT), which are designed to ensure a tight face-to-respirator seal for each specific wearer’s facial characteristics.

The early shortage of N95 respirators resulted from limitations on the required raw materials, limited capacity to manufacture respirators, and the ability of supply and distribution chains to handle increased global demand. For this reason, researchers sought to demonstrate the potential for decontamination and reuse of existing N95 respirators. Standardized procedures are well established for the decontamination and reuse of medical equipment, such as autoclaving, steam treatment, and chemical inactivation (e.g., bleach) (1). Decontamination of most medical equipment is verified using spore-based biological indicators (2). Unlike most hospital equipment (e.g., steel, metal, plastic) or fabrics (e.g., blankets), for which standardized decontamination methods exist (1), N95 respirators are generally not intended for reuse (3). Thus, many of the standard decontamination approaches deform, damage, or destroy the integrity of N95 respirator fabric, nosepiece materials, or elastic straps (4–8). Hydrogen peroxide (H_2_O_2_)-based methods have been successfully adapted for use in decontamination of N95 respirators (4, 6–22), with indications that these methods are less damaging than other decontamination methods (e.g., chemical or steam) and can penetrate the densely woven fabric of respirator facepieces (4, 6–8, 14, 22). In addition, the virucidal capability of H_2_O_2_ decontamination methods has been previously demonstrated (23–25). Vapor-phase H_2_O_2_ methods (e.g., VHP and other patented methods) have been used to decontaminate N95 respirators and were granted temporary U.S. Food and Drug Administration (FDA) emergency use authorization (EUA) for health care use during the pandemic (26). However, these methods employ high concentrations of hydrogen peroxide (30 to 70%) and elevated temperature to achieve vaporization. Vapor-phase H_2_O_2_ (VHP) methods at these concentrations may pose increased health risks to decontamination personnel and, combined with elevated temperature, can result in notable respirator material decay (27). Historical aerosolized H_2_O_2_ (aHP) methods utilized lower peroxide concentrations (5 to 6%) with silver ions and other antimicrobial agents, and activated aerosolization via plasma, nozzle pressure, or ultrasound, with similar limitations as VHP methods (28–32). These methods have not received strong comparative support in U.S. markets due to lower decontamination effectiveness (30–38). Therefore, we utilized a recently developed aHP method (i.e., the CURIS system) which dispenses a low-concentration hydrogen peroxide solution through a precision adjustable nozzle (39). The unit design and decontamination process characteristics allow consistent distribution of disinfectant over time and enable effective decontamination of space and materials. The aHP approach has the potential to scale up for large clinical settings, since the number of respirators that can be decontaminated simultaneously is limited only by the room size.

At present, respirator manufacturers have not approved protocols for N95 respirator decontamination (3). To address immediate pandemic needs, health care settings have referenced U.S. Centers for Disease Control and Prevention (CDC) guidelines on provisional N95 respirator decontamination and reuse (40, 41). Battelle Memorial Institute of Columbus, Ohio, received FDA EUA status for an N95 decontamination protocol using a VHP method based on a prior study addressing the potential for respirator reuse in emergency scenarios (4, 26). This study included evaluation of respirator structure, filtration, and manikin fit-testing and used bacterial spore-based biological indicators to demonstrate effective decontamination (4); however, viral inactivation testing and respirator fit-testing on live subjects were not performed. Since the current pandemic involves a respiratory viral pathogen, multiple decontamination protocols for N95 respirators have been actively investigated at research universities and medical centers (6–21).

Given the reduced personnel health risks of using aHP, we assessed the ability of an aHP decontamination protocol to achieve viral and microbial decontamination of N95 respirators while preserving respirator fit and integrity over multiple treatment cycles. Several respirator models in use by local health care and research personnel were included. Viral decontamination was tested using multiple species representing a range of pathogen characteristics: Pseudomonas phi6 bacteriophage (phi6), herpes simplex virus 1 (HSV-1), coxsackievirus B3 (CVB3), and SARS-CoV-2. Commercial Geobacillus stearothermophilus spore-based biological indicators were used in parallel throughout the aHP process to verify effectiveness of decontamination. We measured the inactivation of viruses by passive drying and by active aHP decontamination. Fitness of respirators for reuse was assessed by qualitative and quantitative respirator fit-testing after decontamination, including up to 10 cycles of aHP treatment. Respirator structure and filtration efficiency testing was also performed. We also acquired real-time and diffusion sampler analyses of hydrogen peroxide levels throughout the decontamination process to monitor user safety.

## RESULTS

This study was intended to rigorously evaluate a protocol for decontamination and reuse of N95 respirators using aerosolized hydrogen peroxide (aHP) treatment (7% H_2_O_2_; CURoxide). The N95 respirator facepiece models examined here represent those frequently used at Penn State or within the Penn State Health system. Six N95 respirator models were selected, with the greatest number available being the 3M 8511 model (Table 1, Fig. S1 in the supplemental material). The decontamination process was performed in the BSL3 enhanced facility, enabling the assessment of viral inactivation across multiple biosafety levels, including SARS-CoV-2 (Table 2, Fig. S2). The BSL3 facility employs aHP on a routine basis to decontaminate solid equipment, and we adapted this protocol to account for the absorbent nature of N95 respirators. Standard aHP charge, pulse, and dwell period parameters were adjusted to optimize cycle times (Table 3) and account for biological indicator and virology results. Our final aHP process (matched to room size) utilized an 11:43 charge period to establish aHP, followed by six pulse charges evenly spaced over 30 min to maintain H_2_O_2_ concentrations and a 20-min dwell period (Table 3).

**TABLE 1:** N95 respirator facepiece models included in this study

Brand and model	No. in study	Style	Type	Exhalation valve	Notes
3M 8511	77	Molded	Nonsurgical	Yes	
3M 1860	10	Molded	Surgical	No	
3M 1870+	11	Folded	Surgical	No	Highest fluid resistance^*a*^
3M 9211+	12	Folded	Nonsurgical	Yes	Same fabric as 1870+
Honeywell Sperian N11125	5	Molded	Nonsurgical	Yes	
Alpha Pro Tech	65	Folded	Surgical	No	

a Highest level of fluid resistance according to ASTM F1862 at 160 mm Hg (42).

**TABLE 2:** Characteristics of virus species used to test inactivation by aerosolized H2O2 compared to SARS-CoV-2

Virus species (abbreviation, taxonomic family)	Diam (nm)	Capsid/virion shape	Genome type, ~size^*a*^	Titer inactivated by aHP (PFU/mL)^*b*^	Biosafety level (BSL)
Severe acute respiratory syndrome coronavirus 2 (SARS-CoV-2, *Coronaviridae*)	120	Enveloped, no icosahedral capsid	Linear (+) ssRNA genome, ~30 kbp	1.6 × 10^5^	BSL3
Herpes simplex virus 1 (HSV-1, *Herpesviridae*)	200	Enveloped, icosahedral	Linear dsDNA genome, ~152 kbp	2.0 × 10^6^	BSL2
Coxsackievirus B3 (CVB3, *Picornaviridae*)	30	Nonenveloped (naked), icosahedral	Linear (+) ssRNA genome, ~7.4 kbp	5.9 × 10^4^	BSL2
Pseudomonas phi6 bacteriophage (phi6, *Cystoviridae*)	85	Enveloped, icosahedral	Segmented, dsRNA genome, ~13.3 kbp	2.4 × 10^8^	BSL1

a ds, double-stranded; ss, single-stranded; ssRNA genomes are either (+) positive or (–) negative sense.

b Each viral species was tested for decontamination at the maximum available titer.

**TABLE 3:** Optimization of aHP treatment parameters

aHP cycle no.	Room vol (ft^3^)	Charge period	Pulse period	Dwell period	aHP parameter set
1a	1,700	16:20	40:00	0:00	Initial
1b	1,700	16:20	40:00	0:00	Initial
2	1,700	11:43	30:00	0:00	Modification 1
3	1,700	11:43	30:00	0:00	Modification 1
4	1,700	11:43	30:00	0:00	Modification 1
5	1,700	11:43	30:00	20:00	Final
6	1,700	11:43	30:00	20:00	Final
7	1,700	11:43	30:00	20:00	Final
8	1,700	11:43	30:00	20:00	Final
9	1,700	11:43	30:00	20:00	Final
10	1,700	11:43	30:00	20:00	Final
11	1,700	11:43	30:00	20:00	Final
12	1,700	11:43	30:00	20:00	Final
Post 1	1,840	12:41	30:00	20:00	Final^*a*^
Post 2	1,840	12:41	30:00	20:00	Final^*a*^
Post 3	1,840	12:41	30:00	20:00	Final^*a*^

a These cycles were carried out in a larger room, which necessitated adjustment of the charge time to account for the larger room volume.

10.1128/msphere.00303-22.2 FIG S1 Respirator facepiece models and characteristics. (A) 3M models 1860 and 8511 (left) have a molded facepiece, with 8511 including an exhalation valve. (B) Virus-inoculation via droplets spotted onto the respirator fabric are shown for two examples. (C) 3M models 1870+ and 9211+ share a common folded fabric model, with a high fluid resistance of the fabric. 3M 9211+ includes an exhalation valve. Several of the facepieces (1860, 1870+, 9211+) display the metal grommet from insertion of the probe used for quantitative fit testing (QNFT). The inner face of each respirator is shown, which for the models shown in panel C required a prop to hold them open. Download FIG S1, PDF file, 0.9 MB. Copyright © 2022 Derr et al. 2022 Derr et al. https://creativecommons.org/licenses/by/4.0/ This content is distributed under the terms of the Creative Commons Attribution 4.0 International license.

10.1128/msphere.00303-22.3 FIG S2 Respirator staging and CURIS unit. (A) Respirator staging (partial loading) on metal rack with binder clips and rack centrally positioned in the prep room. (B) Prep room door sealed with polyethylene sheeting and nonporous adhesive tape to prevent migration of H_2_O_2_ outside the prep room. (C) Indelible ink pen hash marks (arrows) placed to notate completed aHP cycle, at time of cycle completion, or collection/transport for subsequent fit-testing or virus inactivation testing. (D) CURIS decontamination unit with adjustable aHP dispersal nozzle and programmable inputs. Download FIG S2, PDF file, 0.6 MB. Copyright © 2022 Derr et al. 2022 Derr et al. https://creativecommons.org/licenses/by/4.0/ This content is distributed under the terms of the Creative Commons Attribution 4.0 International license.

### Chemical verification and biological validation of the aerosolized H_2_O_2_ process.

Chemical indicators and bacterial spore-based biological indicators (BIs) were used to verify aHP treatment and decontamination. All chemical indicators located throughout the treatment area (prep room) during all aHP cycles confirmed exposure to hydrogen peroxide. For cycles in which charge, pulse, and dwell periods were utilized, all BIs similarly indicated successful decontamination, with the exception of aHP cycles 3 and 5 (Table 4, Fig. S3). In cycle 3 the additional dwell period was not yet implemented, and BIs indicated an unsuccessful decontamination cycle (1 positive, 5 negative). External contamination of one spore coupon after treatment cycle 5 (via dropping) likely resulted in the single positive indicator for this cycle (Table 4). Overall chemical and biological indicator results indicated successful aHP treatment and decontamination of N95 respirators.

**TABLE 4:** Spore-based biological indicator (BI) and virus inactivation results

aHP cycle no.	aHP-treated spore-based BIs	Control spore-based BIs	Spore-based BI results	aHP parameter set	Viruses tested^*a*^	Virus inactivation results
1a	12	1	Pass	Initial		
1b	12	1	Pass	Initial		
2				Modification 1		
3	6	1	Fail (1 of 5 positive)	Modification 1	phi6, HSV1, CVB3	3 of 64 sites positive
4	6	1	Pass	Modification 1		
5	6	1	Fail^*b*^ (1 of 5 positive)	Final		
6	6	1	Pass	Final	HSV1, CVB3	1 of 26 sites positive
7	6	1	Pass	Final		
8	6	1	Pass	Final		
9	6	1	Pass	Final		
10	6	1	Pass	Final		
11	6	1	Pass	Final		
12	6	1	Pass	Final		
Post 1				Final	phi6, HSV1, CVB3	0 of 66 sites positive
Post 2	12	1	Pass	Final	SARS-CoV-2	0 of 24 sites positive
Post 3	12	1	Pass	Final	SARS-CoV-2	0 of 24 sites positive

a The N95 respirators used for virus inoculation at each cycle were subjected to all preceding aHP cycles; e.g., respirators used for viral testing in aHP cycle no. 6 had been through 5 prior aHP cycles. For cycles labeled “post,” the respirators for viral testing had been through 10 to 12 prior aHP cycles.

b A spore disc dropped during transfer to medium was the suspected cause of this failure.

10.1128/msphere.00303-22.4 FIG S3 Spore-based biological indicators (BIs). (A) Commercial BIs (Steris Spordex) containing Geobacillius stearothermophilis spores are sold on discs in Tyvek/glassine envelopes with accompanying vials of culture medium. After treatment, spore discs are transferred to culture medium and incubated at 55°C for at least 7 days post-decontamination cycle. (B) Medium color change to yellow indicates bacterial growth, as seen in the positive-control tube on the far right. In addition to placement of BIs around the treatment room (see Materials and Methods for details), BIs were nested between two molded respirators (3M 1860) and taped together (C) and were enclosed within a flat-fold type respirator (3M Aura 9211+; not pictured). All BIs positioned in this manner were rendered inactive after aHP treatment. Download FIG S3, PDF file, 0.1 MB. Copyright © 2022 Derr et al. 2022 Derr et al. https://creativecommons.org/licenses/by/4.0/ This content is distributed under the terms of the Creative Commons Attribution 4.0 International license.

### Real-time hydrogen peroxide monitoring.

Real-time measurement of H_2_O_2_ concentrations during decontamination were obtained with a portable, real-time Analytical Technology, Inc. (ATI) PortaSens II monitor (Table 5). H_2_O_2_ concentrations in the treatment area were measured at ≥120 ppm during the charge and pulse periods (maximum sensor capability). Hydrogen peroxide concentrations outside the sealed entryway were low or undetected (0 ppm). On occasions when a broken seal arose in the tape around the door (Fig. S2), low concentrations were detectable outside the door (up to 3 ppm). Once corrected, these levels immediately dropped to 0 ppm. During aeration after aHP treatment, H_2_O_2_ levels were monitored until concentrations measured ≤2 ppm. At this time, research personnel entered the prep room to measure H_2_O_2_ concentrations on respirator surfaces. Hydrogen peroxide concentrations were observed to decline rapidly during aeration within 20 to 30 min of door opening (Table 5). Initial respirator surface concentrations (on fabric) typically exceeded 2 ppm. Subsequent respirator drying to achieve <1 ppm required further room aeration (exhaust ventilation) exceeding 2 h or overnight (Table 5). Once H_2_O_2_ respirator surface concentrations measured <1 ppm, they were either subjected to further cycles of aHP or collected for fit-testing or virus inactivation analysis.

**TABLE 5:** Portable real-time H_2_O_2_ measurements, using ATI PortaSens II

aHP cycle no.	Measurement location^*a*^	Measurement condition	H_2_O_2_ concn or range (ppm)^*b*^	Time period to decayed concn (minutes)	aHP parameter set
1a	IC, wall port	Charge	>120		Initial
1a	IC, wall port	Aeration, to entry	2.5–57	52	Initial
1b	IC, wall port	Charge	>120		Initial
2	No data	No data	No data	No data	Modification 1
3	IC, door open	Aeration, to entry	1.5–11.6	20	Modification 1
3	IC, door open	Aeration, to respirator measurement	1.5–0.8	40	Modification 1
4	IC, door open	Aeration, to entry	2–10.6	20	Modification 1
4	Respirator surface	After overnight drying	0–3	Overnight	Modification 1
5	IC, wall port	Charge	>120		Final
6	IC, wall port	Pulse	120		Final
6	respirator surface	After overnight drying	0	overnight	Final
7	IC, wall port	Charge	>120		Final
7	OC, seal check(s)	Charge, pulse, dwell	0		Final
7	IC, door open	Aeration, to entry	2–7	27	Final
7	Respirator surface	After overnight drying	0	Overnight	Final
8	IC, wall port	Pulse	88		Final
9	IC, wall port	Charge	>120		Final
9	IC, door open	Aeration, to entry	1.6–24	20	Final
9	Respirator surface	Aeration, to respirator measurement	<1	85	Final
10	OC, seal check	Pulse, door seal repair	0–1.6		Final
10	IC, door open	Aeration, to entry	1.3–15	32	Final
10	Respirator surface	After overnight drying	<1	Overnight	Final
11	IC, door open	Aeration, to entry	1–13.6	35	Final
11	Respirator surface	After overnight drying	<1	Overnight	Final
Post	Respirator surface	Aeration to 1 ppm, respirator data log	1–8.1	150	Final

a IC, inside containment/treatment area; wall port, through sealable wall port; door open, door ajar after door seal removed; OC, outside containment/treatment area; seal check, prep room door tape seal; respirator surface, at respirator surface following room entry.

b A low measurement value represents the lowest concentration following concentration decay; a high value represents starting concentration (prior to decay).

### Qualitative and quantitative fit-testing after aHP decontamination.

**FIG 1:**
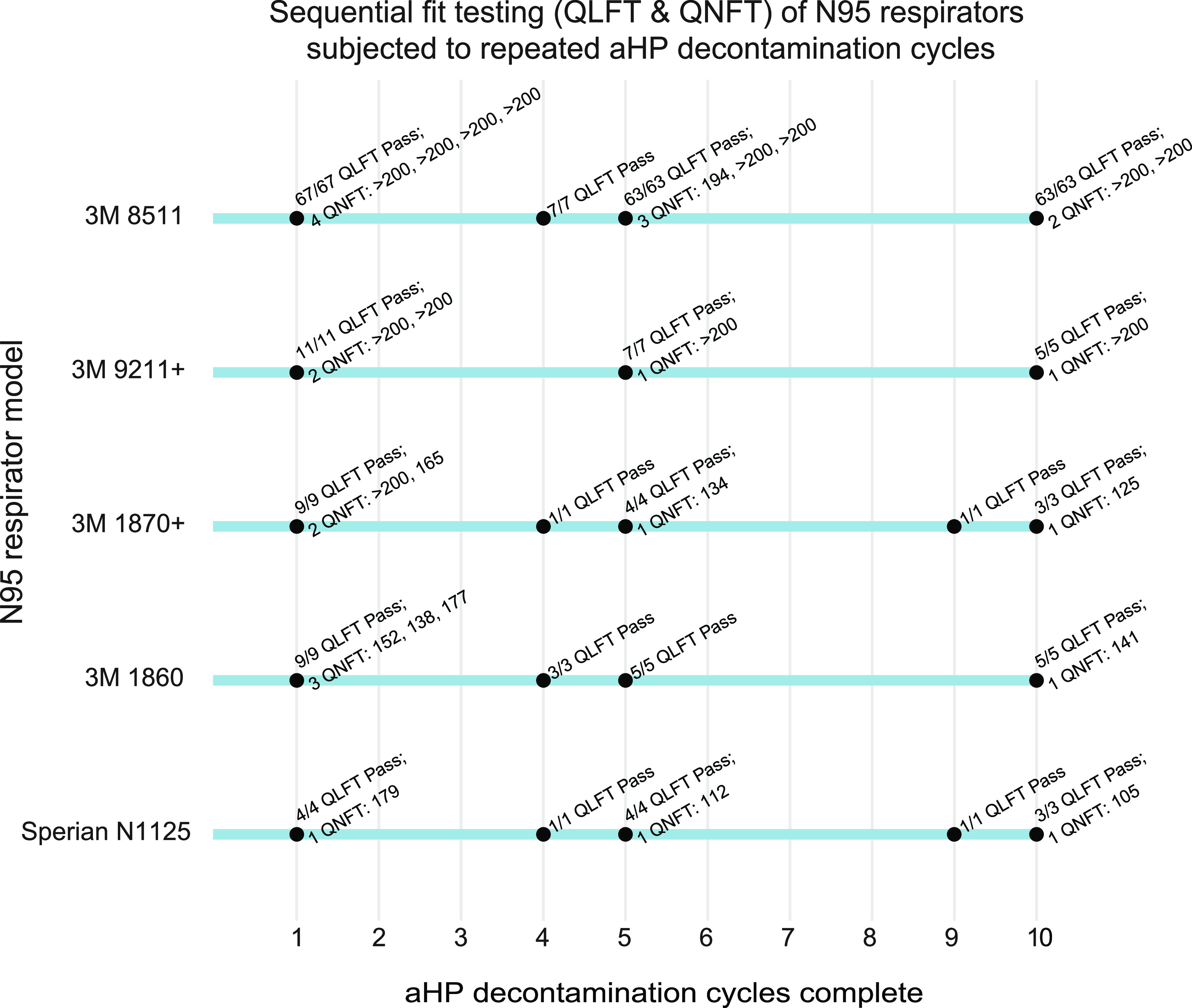
Sequential fit testing (QLFT and QNFT) of N95 respirators subjected to repeated aHP decontamination cycles. Results demonstrate that all 3M model 8511 respirators successfully pass QLFT and QNFT after 1, 5, and 10 cycles. In particular, 8 of 9 QNFT results for the 3M model 8511 respirator surpassed a fit factor of 200, the maximum reportable by the test method, providing qualitative, yet objective, evidence of the safety margin related to fit integrity after 10 aHP decontamination cycles. All respirator models except Alpha Pro Tech (which had inconsistent fit-test results; see Materials and Methods for details) passed all QLFT and QNFT to which they were subjected. Model Alpha Pro Tech (aHP cycle 1) was only included for a single cycle and thus is not shown here.

We employed both qualitative (QLFT) and quantitative (QNFT) fit-testing. QLFT allowed repeated nondestructive testing across multiple rounds of decontamination, while QNFT provided numerical data on fit factor and ruled out any false negatives due to user fatigue. QLFT and QNFT was conducted after the first, fifth, and tenth decontamination cycles for both a male and female subject. These tests included a large number of 3M model 8511 respirators (for which we had the most available in the starting pool) and a smaller number of 5 other respirator models (Table 1, Fig. 1). All respirators passed all QLFT and QNFT (Fig. 1). One respirator facepiece (3M model 1870+) experienced a broken strap after the eighth cycle of aHP; this respirator had passed QLFT successfully in earlier cycles but was excluded from final QNFT. No other failures occurred; therefore, with 95% confidence, at least 95% of 3M model 8511 respirators maintained a successful fit seal after 10 aHP decontamination cycles. QNFT results with 3M model 8511 respirators passed the minimum passing fit factor of 100 and the maximum quantifiable fit factor of 200(+) in 8 of 9 tests. The numerical range of passing fit factors (100 to 200), and available sample quantity, limit any further statistical analysis. The successful fit-testing results indicate that repeated cycles of aHP decontamination do not interfere with respirator fit for reuse.

### Respirator filtration efficiency testing.

N95 respirator filtration efficiency testing was conducted on aHP-treated 3M 8511 respirators using the NIOSH test protocol under full-load test conditions (Table 6). No visible degradation was found on inspection (e.g., metal nose guard discoloration, unusual thinning, or wear). Filtration efficiencies were found to exceed 95% for all eight respirators subjected to 10 cycles of aHP decontamination. Therefore, for at least 10 aHP cycles, there were no adverse impacts or loss of N95 respirator efficiencies. Two additional unused, untreated 3M 8511 respirators were additionally tested and found to have slightly reduced filtration efficiencies (94.4%, 94.6%), consistent with the respirator manufacturer’s 5-year shelf-life limitation. These data indicate that overall respirator performance was maintained over time and after aHP treatment.

**TABLE 6:** Respirator filtration efficiency testing results following 10 cycles of aerosolized hydrogen peroxide decontamination

Full-loading efficiencies—3M model 8511 (N95 respirator)^*a*^						
Sample ID^*b*^	Initial test flow rate (LPM)^*c*^	Initial test resistance (mm H_2_O)	Initial test penetration (%)	Maximum penetration (%)	Filter efficiency^*d*^ (%)	Result
MS-1	84	6.5	0.67	2.29	97.71	Pass
MS-2	85	6.7	0.82	2.38	97.62	Pass
MS-3	84	6.9	1.46	4.16	95.84	Pass
MS-4	85	6.8	1.75	4.14	95.86	Pass
FS-1	85	7.1	2.02	4.58	95.42	Pass
FS-2	84	6.6	1.47	3.5	96.50	Pass
FS-3	85	6.6	0.20	2.37	97.63	Pass
FS-4	86	7.0	1.47	4.12	95.88	Pass
Test specification	81–89			≤5.0	≥95.0	

a Respirator testing analysis performed by ICS Laboratories of Brunswick, OH.

b MS, FS, (*x*), male subject or female subject sample respirator ID.

c LPM, liters per minute.

d Filter efficiency percentage is based on maximum penetration value.

### Application of multiple viral species to N95 respirator facepieces.

Viral inactivation on respirator facepieces was anticipated to occur both by the passive process of drying or desiccation, and by the active process of aHP decontamination. Multiple virus species were included to test the decontamination potential of aHP against viruses in general, as well as against SARS-CoV-2. These included phi6, HSV-1, CVB3, and SARS-CoV-2 (see Table 2 and Methods for details). Respirators used for virus inactivation testing were those previously subjected to aHP treatment and fit-testing, to spare overall respirator consumption (see Table 4 and Materials and Methods). Initial application of virus to different respirator facepiece types revealed clear differences in relative absorption versus fluid repulsion (Fig. S1). 3M respirator models 1870+ and 9211+ have a common outer fabric which is listed by the manufacturer as having the highest fluid resistance of any N95 respirator (Table 1) (42). In our testing, these two respirator models displayed no apparent absorption of virus inoculum and instead dried with a “coffee ring effect.” All other respirator types (Table 1) experienced a combination of liquid spreading, absorption, and evaporative drying of the virus inoculum droplet. Viruses were inoculated onto different areas of each respirator facepiece model, including the outer and inner fabric surfaces, the elastic strap, and where present, the inner and outer surface of the plastic exhalation valves (see Table S1 for specific sites and respirator models).

### Decontamination of virus-inoculated respirators by aerosolized H_2_O_2_ treatment.

**FIG 2:**
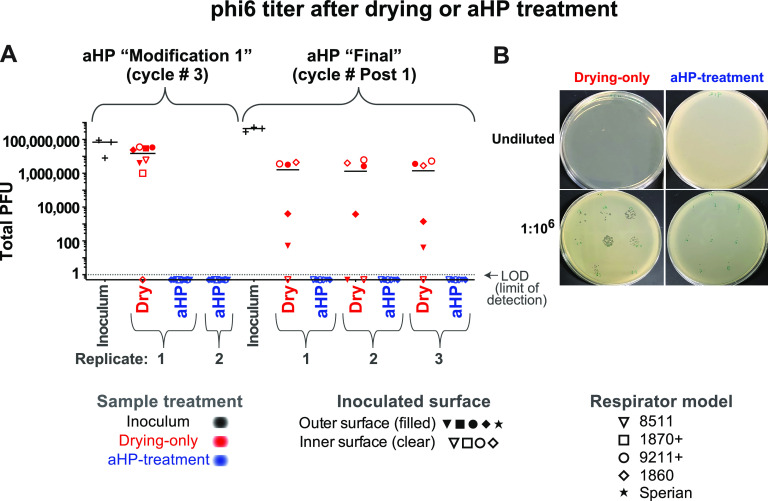
Infectious titer of phi6 bacteriophage inoculated on N95 respirator facepieces was eliminated after aerosolized H_2_O_2_ (aHP) decontamination. (A) Data are plotted for each aHP cycle in which viral testing was done (see Table 4). Multiple models of N95 respirator (see Table 1) were inoculated and either treated as drying-only controls (red) or subjected to aHP treatment (blue). The respirator surface and model are indicated by the symbol shape and fill. The median of all points within a given aHP cycle and treatment is indicated by a solid horizontal line. The dashed horizontal line indicates the limit of detection (LOD) at 1 viral PFU in the resuspended but undiluted volume from the site of viral inoculation. (B) Petri dish plating of bacterial lawns exposed to phi6 from drying-only (left side) or aHP-treated (right side) respirator inoculation sites. These were applied to the bacterial lawn either as an undiluted resuspension (top row) or 1:10^6^ dilution applied to focal points (bottom row). For the purposes of illustrating the decontaminated sites where zero plaques were detected, these numbers were replaced with fractional values (0.5), to allow their visualization on this log-scale plot. See Table S1 for all data values.

**FIG 3:**
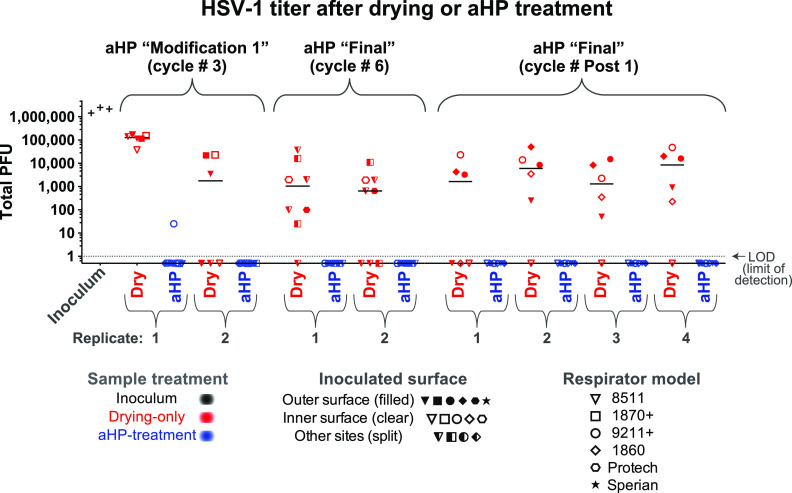
The infectious titer of HSV-1 inoculated on N95 respirator facepieces was reduced by drying and eliminated after aerosolized H_2_O_2_ (aHP) decontamination. Data are plotted for each aHP cycle in which viral testing was done (see Table 4). For HSV-1, the sole positive plaque after aHP treatment occurred in aHP cycle no. 3, when the modification 1 parameters were in use (Tables 3 and 4). This failure, in concert with a spore-based biological indicator and 2 CVB3 plaques (Fig. 4), motivated the addition of a dwell time in the final aHP parameters. As in Fig. 2, multiple models of N95 respirator (see Table 1) were inoculated and either treated as drying-only controls (red) or subjected to aHP treatment (blue). The respirator surface and model are indicated by the symbol shape and fill. The median of all points within a given aHP cycle and treatment is indicated by a solid horizontal line. The dashed horizontal line indicates the limit of detection (LOD) at 1 viral PFU in the resuspended but undiluted volume from the site of viral inoculation. For the purposes of illustrating the decontaminated sites where zero plaques were detected, these numbers were replaced with fractional values (0.5) to allow their visualization on this log-scale plot. See Table S1 for all data values.

**FIG 4:**
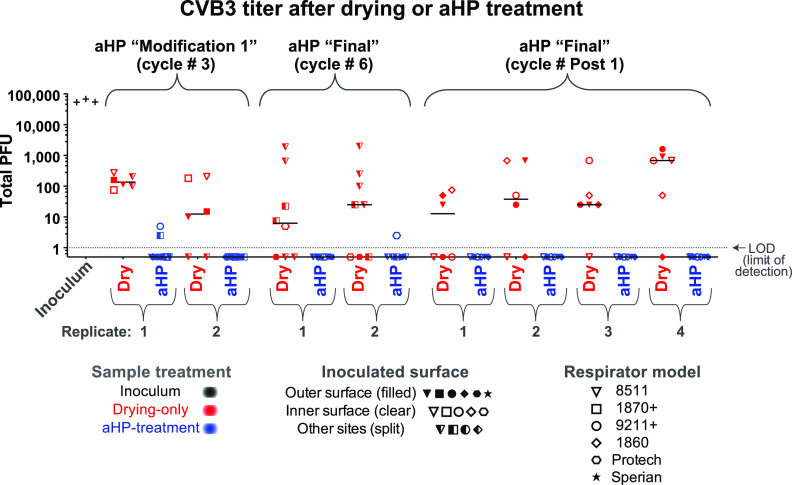
The infectious titer of CVB3 inoculated on N95 respirator facepieces was reduced by drying and eliminated after aerosolized H_2_O_2_ (aHP) decontamination. Data are plotted for each aHP cycle in which viral testing was done (see Table 4). For CVB3, two positive plaques after aHP treatment occurred in aHP cycle no. 3, when the modification 1 parameters were in use (Tables 3 and 4). This failure, in concert with a spore-based biological indicator and 1 HSV-1 plaque (Fig. 3), motivated the addition of a dwell time in the final aHP parameter. The only other positive CVB3 plaque after aHP treatment occurred in aHP cycle 6, and no plaques were detected in the replicate or in parallel samples. As in Fig. 2, multiple models of N95 respirator (see Table 1) were inoculated and either treated as drying-only controls (red) or subjected to aHP treatment (blue). The respirator surface and model are indicated by the symbol shape and fill. The median of all points within a given aHP cycle and treatment is indicated by a solid horizontal line. The dashed horizontal line indicates the limit of detection (LOD) at 1 viral PFU in the resuspended but undiluted volume from the site of viral inoculation. For the purposes of illustrating the decontaminated sites where zero plaques were detected, these numbers were replaced with fractional values (0.5) to allow their visualization on this log-scale plot. See Table S1 for all data values.

**FIG 5:**
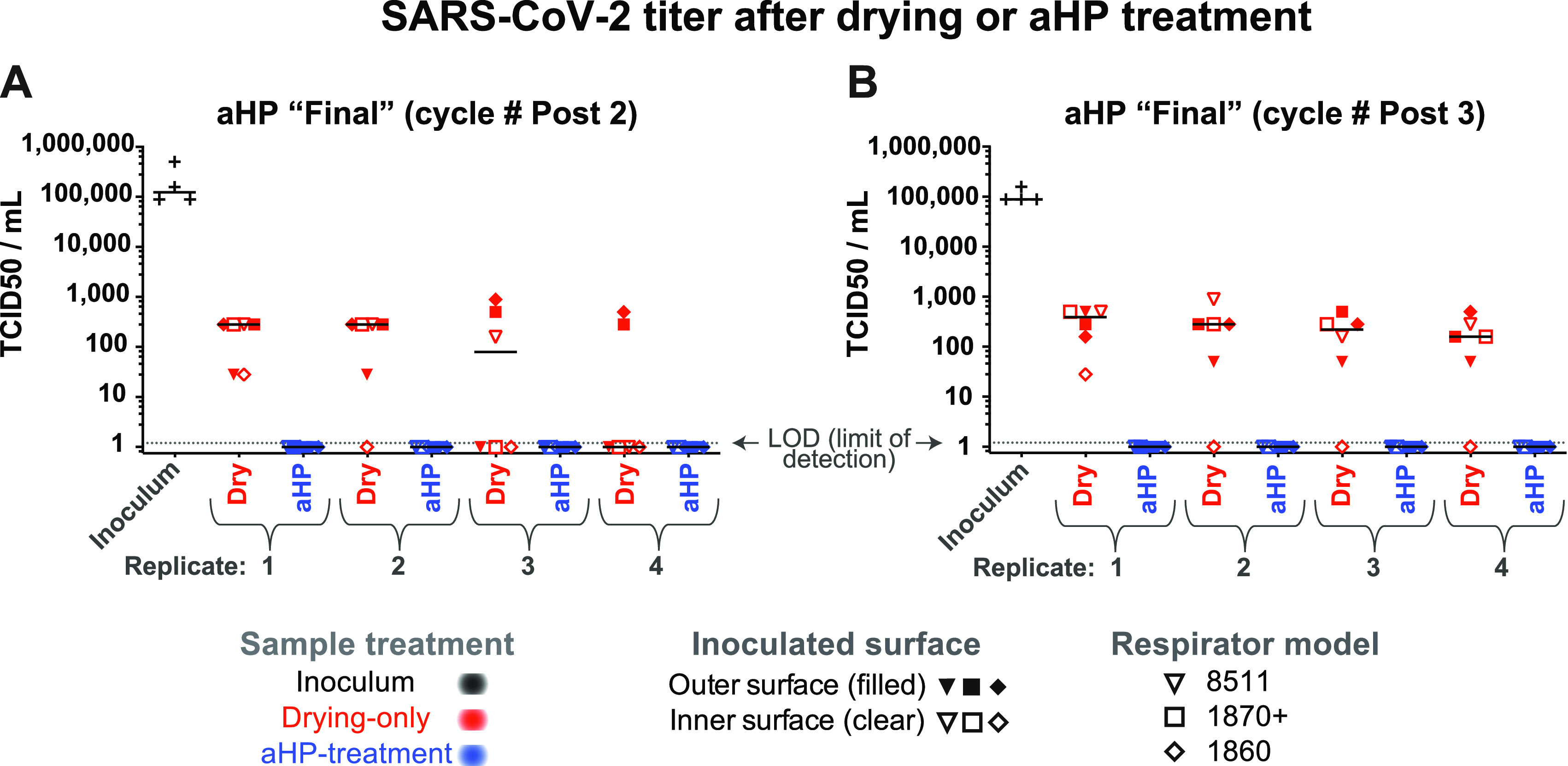
The infectious titer of SARS-CoV-2 inoculated on N95 respirator facepieces was reduced by drying and eliminated after aerosolized H_2_O_2_ (aHP) decontamination. (A and B) Data are plotted separately for each aHP cycle in which viral testing was done (see Table 4). For SARS-CoV-2, no infectious virus was detected by TCID_50_ assay after aHP treatment. As in Fig. 2, multiple models of N95 respirator (see Table 1) were inoculated and either treated as drying-only controls (red) or subjected to aHP treatment (blue). The respirator surface and model are indicated by the symbol shape and fill. The median of all points within a given aHP cycle and treatment is indicated by a solid horizontal line. Viral titer was determined by 50% tissue culture infectious dose (TCID_50_) assay in 96-well plates, with a limit of detection (LOD) of 1.2 (see Materials and Methods for details). For the purposes of illustrating the decontaminated samples where no virus was detected, these numbers were plotted as a value of 1. See Table S1 for all data values.

We set out to assess the effectiveness of aHP treatment for active decontamination of virus inoculated onto N95 respirators. Virus-inoculated respirators were subjected to aHP treatment, using both “modification 1” and “final” parameters (Table 3). Viral testing of decontamination was conducted during five independent aHP cycles (Table 4), using the maximum inoculum titer available for each viral stock preparation (Table 1). For phi6 bacteriophage, this included 34 “aHP-treated” sites spanning two independent rounds of testing (Fig. 2). For HSV-1 and CVB3, this included 62 and 60 aHP-treated sites, respectively, spanning three independent rounds of testing (Fig. 3 and 4). Across a total of 204 respirator sites inoculated with one of four virus species tested (Table 2; see also Table S1), only four sites had any detectable virus remaining. Three of these rare positive virus plaques were detected in an aHP cycle using the modification 1 parameters (Fig. 3 and 4; Table 4; see also Fig. S4); these were a key motivation to add the dwell time for the final aHP cycle parameters (Table 3). Overall, aHP treatment produced a 4 to 7 log_10_ reduction in viral load (10^7^ reduction for phi6, Fig. 2; 10^5^ reduction for HSV-1, Fig. 3; 10^4^ reduction for CVB3, Fig. 4). There was no observable difference in the effectiveness of aHP decontamination for inner versus outer surfaces of respirators (Fig. 2-5; see also Table S1) or in limited testing of alternative inoculation sites such as elastic straps (Fig. 3 and 4, Table S1). The success of virus inactivation by aHP treatment mirrored the results of spore-based biological indicators (Table 4).

10.1128/msphere.00303-22.5 FIG S4 Viral titration demonstrates inactivation and loss of infectious units due to drying and aerosolized H_2_O_2_ (aHP) decontamination. Shown here are representative examples of serial dilutions (titration) of HSV-1 or CVB3 that were spotted onto 3M 9211+ respirators and then resuspended and plated onto monolayers of Vero detector cells. Viral plaques, which are visible as clear foci of infection (PFU) on the background of methylene blue-stained cells, were visualized at 72 h postinfection. The plate shown in panel A illustrates serial dilution of a high concentration of HSV-1 after drying only (10^5^ PFU; see plot in Fig. 3) and a lower concentration of CVB3 after drying only (10^2^ PFU; see plot in Fig. 4). The plate shown in panel B is from an aHP cycle run with modification 1 parameters, when no dwell time was used (aHP cycle 3; see Tables 3 and 4), and commercial spore-based biological indicators indicated a failure of decontamination. Even on this partial aHP decontamination, this sensitive detection method revealed only three wells (two of which are shown above) with any viral plaques (indicated by arrowheads; these equate to 25 PFU of HSV-1 and 5 PFU of CVB3; see plots in Fig. 3 and 4). Download FIG S4, PDF file, 0.6 MB. Copyright © 2022 Derr et al. 2022 Derr et al. https://creativecommons.org/licenses/by/4.0/ This content is distributed under the terms of the Creative Commons Attribution 4.0 International license.

10.1128/msphere.00303-22.6 TABLE S1 aHP viral testing values. Excel file of data underlying Fig. 2, 3, 4 and 5 and Table 4. These include the number of PFU for phi6, HSV-1, and CVB3, as well as TCID^50^(log10)/mL values for SARS-CoV-2. As noted in Fig. 2, 3, 4 and 5, for samples where no virus was detected, these numbers were replaced with nonzero values to allow their visualization on the log-scale plots in Fig. 2, 3, 4 and 5. For HSV-1, CVB3, and phi6, the “no virus detected” values were set to 0.5 (less than 1 plaque detected), and for SARS-CoV-2, the values were set to 1 (less than the limit of detection of 1.2 TCID_50_[log10]/mL). Download Table S1, XLSX file, 0.02 MB. Copyright © 2022 Derr et al. 2022 Derr et al. https://creativecommons.org/licenses/by/4.0/ This content is distributed under the terms of the Creative Commons Attribution 4.0 International license.

### Inactivation of multiple viral species by air-drying on respirators.

Since passive viral inactivation by drying likely occurs during the time involved in decontamination, we also measured the amount of virus remaining on inoculated but untreated N95 respirators. For each cycle of viral testing, the duration of passive viral inactivation was matched to the duration of the decontamination process (including aHP treatment, subsequent aeration, and transport). These “drying-only” samples confirmed a partial loss of viral infectiousness, ranging from 10- to 100-fold for phi6 and HSV-1 and 100-fold or greater for CVB3 and SARS-CoV-2 (Fig. 2-5; see also Fig. S4 and Table S1). For phi6 bacteriophage, this included 26 drying-only inoculation sites spanning three independent rounds of testing (Fig. 2). For HSV-1 and CVB3, this included 52 drying-only inoculation sites for each virus species, spanning three independent rounds of testing (Fig. 3 and 4, respectively). Viral inactivation by drying was not markedly different on inner versus outer surfaces of the respirator models (Fig. 2-5; see also Table S1) or in limited testing of alternative inoculation sites, such as elastic straps or plastic exhalation valves (Fig. 3, Fig. 4, Table S1).

### Inactivation of SARS-CoV-2 by aHP treatment.

Experimental testing of SARS-CoV-2 by aHP necessitated that all work be completed at BSL3. These studies were conducted with the 3M 8511, 1860, and 1870+ N95 respirators and included two independent rounds of testing (Fig. 5). As with the surrogate virus species, SARS-CoV-2 was first inoculated onto respirator facepieces. The drying-only controls were left in the BSL3 ambient environment, while matched samples were subjected to aHP treatment. The respirators used for SARS-CoV-2 testing had been subjected to 10 prior rounds of aHP before virus inoculation (Table 4). For SARS-CoV-2, viral testing included 48 drying-only inoculation sites and 48 aHP-treated sites, spanning two independent rounds of testing (Fig. 5). We observed a partial loss of viral infectiousness for SARS-CoV-2 due to drying (e.g., input of 10^6.125^ 50% tissue culture infective dose [TCID_50_]/mL versus ~10^2^ TCID_50_/mL after drying; Fig. 5, see also Table S1). Importantly, no infectious SARS-CoV-2 remained on any respirator model after aHP decontamination (Fig. 5, Table S1).

## DISCUSSION

Based on a series of 10 respirator decontamination cycles and multiple rounds of viral inactivation testing, multiple N95 respirator models tested were found to be suitable for aHP decontamination and reuse. We found that respirators successfully passed qualitative respirator fit testing after multiple cycles of the aHP decontamination process and ultimately passed tests indicating no loss in filtration efficiency. Most respirator-reuse studies thus far have either focused on verifying fit-testing after respirator decontamination (6–10, 12, 15, 16, 20) or examined how decontamination approaches inactivate one or more virus species on respirators (6–8, 11, 13, 14, 18–21). A few N95 respirator decontamination studies have combined fit-testing with measures of viral inactivation (6–8), but none incorporated viral- or fit-testing after 5 or 10 decontamination cycles as done here, or included the parallel use of biological indicators. Only rarely have studies included extended use between multiple rounds of decontamination cycles (10), although this is a key aspect of reuse that warrants further study. Our study is unique in including multiple measures of respirator integrity via fit-testing, as well as filtration efficiency, and robust verification of viral inactivation using BIs, multiple surrogate viruses, and SARS-CoV-2 in parallel (22). The breadth of this study aims to extend its usefulness beyond the current pandemic.

### Respirator resilience for reuse.

Aerosolized H_2_O_2_ decontamination of the N95 respirators used in this study did not indicate any adverse impact on final respirator filtration efficiency, although extended use of respirators between aHP cycles was not possible in this study. We documented effective control of H_2_O_2_ levels, supporting effective pathogen decontamination, with no detectable researcher exposure. These data support and reflect those of parallel studies that have tested respirator fit after decontamination by other forms of hydrogen peroxide vapor (VHP) and/or similar methods (6–10, 12, 15, 16, 20). Further studies will be needed to assess the H_2_O_2_ concentration profile inside containment, using real-time instruments with a wider detection range and automated data logging.

As noted above, most studies that include successful fit-testing and verification of viral inactivation have not pursued this testing across multiple (i.e., 10) cycles of decontamination (6–8, 20). Lab-based conditions such as those used here do not fully reflect clinical use conditions. While respirators were physically stretched between each decontamination cycle as a proxy for donning and doffing, sustained clinical use includes other stressors which may influence fit and performance (e.g., exhaled moisture or perspiration) (10, 16). However, as noted by the CDC, respirators with obvious signs of use (e.g., makeup or patient fluids) should be discarded and not used for decontamination (40, 41). Other studies have explored respirator decontamination in these real-world use scenarios, albeit without including the parallel testing of viral species, BIs, and cycle numbers that were included here (10, 15–17, 20). We anticipate that future studies will address the implementation of aHP-based decontamination in a clinical-use setting.

During this study, one 3M 1870+ respirator suffered a broken rubber strap after eight cycles of aHP, during intercycle strap stretching. The breakage occurred at a point on the strap that corresponded with a penned hash mark (used to denote decontamination cycle; see Fig. S2). There were no other instances of failure for this or any other respirator models. Prior groups have likewise noted strap-based failures after multiple cycles of respirator decontamination (4, 6, 19, 20). We recommend using care when marking respirators during reuse protocols.

### Viral and biological indicator inactivation.

Viral inactivation by drying depends on multiple factors, including surface type, humidity, temperature, virion size and type, and duration of drying (43–46). All studies of N95 respirator decontamination include both the passive inactivation of viruses by air drying and their active decontamination by aHP or comparable treatment (43–46). Clinical exposure of respirators to SARS-CoV-2 or other viral pathogens would likewise entail ambient drying prior to any respirator decontamination or reuse (e.g., virus may dry onto an N95 in the course of a work shift or during bagging for decontamination or direct reuse). The drying-only time frame used here was shorter than that used in studies modeling reuse in a clinical setting (10, 15–17, 20). This suggests that crisis-capacity protocols that involve respirator reuse after multiple days of drying, even without aHP or active decontamination, likely provide substantial levels of viral inactivation (40, 41).

To model diverse routes of respirator exposure to viral pathogens during use, we inoculated viruses onto different N95 models and surfaces to thoroughly test for the ability of aHP to inactivate viruses. This included different regions of the respirator (e.g., outer versus inner surface) to discern whether any differences in these fabrics would influence viral inactivation. This work was inspired by early efforts to verify viral inactivation in the context of N95 respirator surfaces, such as that of Kenney et al. (13). We found scant evidence of viral survival during the aHP decontamination process. Relative to studies using different decontamination methods to test viral inactivation on N95 respirators, these data show equivalent or better inactivation of viruses (6–8, 11, 13, 14, 18–21, 43).

Commercial bioindicator tests have been relied upon for verification of decontamination for decades (2, 47). Overall, we observed parallel outcomes in terms of successful decontamination of viral species and bacterial spore-based BIs (Table 4), echoing the few other studies that have used these approaches in parallel (14, 18, 20). During establishment of aHP cycle parameters, we noted a concomitant failure of BIs and viral inactivation in cycle 3, before the dwell period was added (Tables 3 and 4). The parallels in success or failure of both BIs and viral inactivation suggest that commercial spore-based BIs provide a useful predictor of success or failure for decontamination of N95 respirators, particularly in settings where direct viral testing is not feasible (2, 10, 15–17). The 10 cycles of aHP decontamination achieved here are well beyond the CDC’s crisis-capacity plans, which recommend no more than five total rounds of respirator reuse (40, 41). While clinical use of respirators, by a multitude of health care workers, was not included in the present study, we foresee that such testing will be an important next step.

We used multiple virus species to test the viral inactivation capabilities of aHP decontamination. These viruses represented multiple characteristics of human viral pathogens, with a range of virion and genome types and sizes, and previously documented environmental stability (44, 45, 48). Like SARS-CoV-1, SARS-CoV-2 has a high level of environmental stability (49–53). Coronaviruses have a lipid-enveloped virion of ~120 nm, with no icosahedral capsid core, containing a single-stranded, linear, positive-sense RNA genome (Table 2). Virions of HSV-1 and phi6 bacteriophage have a lipid envelope, with an underlying icosahedral capsid core. In contrast, CVB3 has a nonenveloped, or naked, icosahedral capsid virus. Prior work has shown that naked-capsid viruses have a higher stability than enveloped viruses, thus demonstrating the range of aHP decontamination abilities (44, 45, 48). While most pathogens utilized here require propagation in mammalian cell lines at biosafety level 2 (BSL2), phi6 can be assayed more flexibly, using rapid bacterial cultures (24-h turnaround) at biosafety level 1. Phi6 is a natural pathogen of the bacterial species Pseudomonas syringae pathovar phaseolicola, which is itself a pathogen of green beans. All viral species examined in this study were effectively decontaminated by aHP (Table 4). While the aerosolization of viruses was not incorporated here, this approach merits inclusion in future studies. Together, the combination of respirator fit testing and virus inactivation testing used here indicate that aHP is a viable decontamination process to enable crisis-capacity reuse of N95 respirators during viral pandemics.

## MATERIALS AND METHODS

### Decontamination facility.

The decontamination process was carried out in the Eva J. Pell Laboratory for Advanced Biological Research at The Pennsylvania State University, University Park campus. This facility is a purpose-built BSL3 enhanced facility, and all required approvals were obtained from the Institutional Biosafety Committee (IBC) for work involving viruses, as described below.

The primary decontamination process was performed within in an approximately 1,700-cubic foot sealed preparation room (prep room), followed by additional virus inactivation testing with SARS-CoV-2 in a separate, nearby 1,840-cubic foot prep room. Procedures and use of personal protective equipment (PPE) suitable for the viruses and materials in use were strictly observed in this biosafety level 3 (BSL-3) facility, which has consistently maintained institutional, CDC, and USDA approval for work with risk group 3 pathogens since its commissioning in 2014.

### Decontamination preparation.

Respirators for decontamination were staged on a portable metal rack located centrally in the prep room (Fig. S2). Filtered and conditioned air was supplied to the prep room, and the air exhausted from the room is HEPA-filtered. Bubble-tight dampers (Camfil Farr) were operated to seal both the supply and exhaust air from associated ductwork during the decontamination cycle. The CURIS decontamination unit was programmed, equipment was positioned, and the room doors were sealed using polyethylene sheeting and nonporous adhesive tape (Fig. S2).

### Decontamination process.

The CURIS decontamination unit programming method utilizes room size to establish the baseline parameters for charge (initial aHP dispensing) and intermittent aHP pulse periods (additional aerosol pulses). This is followed by a user-defined dwell period (when no further aHP is introduced) at closure of the pulse period. Aeration to disperse residual H_2_O_2_ follows the dwell period (i.e., room seals are broken and ventilation resumed). Once the user inputs the room’s cubic volume or dimensions, the CURIS unit calculates a suggested duration of charge and pulse periods. The standard aeration period is 3 h, unless an auxiliary scavenging system or other space aeration system is utilized. To account for absorption of aHP into porous materials in the decontamination space (e.g., N95 respirators), a 30 to 40% increase in these default settings was initially used, as recommended by the manufacturer. Additionally, to ensure adequate contact time of disinfectant to the treated surfaces, a dwell period was added to the continuous and pulse charge periods. Based on initial results, adjustments in the charge, pulse, and dwell periods were made to optimize the decontamination process (Tables 3 and 4).

After completion of the decontamination phase, an aeration or dissipation phase was initiated by removing fixed room seals and opening exhaust dampers for up to 2-h periods, with the room under slight vacuum (0.18 to 0.19″ by water gauge). Adjustments were made to the room air exchange rate during aeration to efficiently dissipate detectable H_2_O_2_ from respirator facepieces. At cycle 5, final parameters were established to include an aeration exhaust rate greater than or equal to 35 air changes/hour, with make-up air supplied by outdoor air. Following this phase, and after room H_2_O_2_ concentration was measured at less than 2 ppm, the respirator-holding rack was either retained under ventilation or transferred to a separate room (referred to as the “finishing room”) with an HVAC air supply curtain to further dry and decompose residual aHP from respirator facepieces to less than 1 ppm H_2_O_2_ (24, 25).

### Respirator handling process.

For respirators subjected to repeated rounds of decontamination as part of this study, decontamination cycles were conducted repetitively from staging through drying. In order to be considered dry or ready for the next cycle, the interior and exterior respirator surfaces were monitored using a calibrated, hand-held real-time H_2_O_2_ monitor (ATI PortaSens II). Once H_2_O_2_ concentrations measured at respirator surfaces at less than 1 ppm, respirators were restaged for the next round of decontamination or packaged and transported for subsequent respirator fit-testing or virus inactivation analysis. Between each cycle, the treated/dried respirators were subjected to manual stress by flexing each respirator bi-directionally and stretching each strap twice, using a hold position similar that used in respirator donning or doffing. A standard thin-line VWR lab marker was to mark each strap for each round of the aHP process (Fig. S2C).

### Spore-based biological indicators.

Commercial biological indicators (BI) were used for verification of decontamination. These commercially prepared spore discs, or “coupons” (Steris Spordex), are enclosed in Tyvek/glassine envelopes (see Fig. S3 for image) and contain a mean spore count of 2.4 × 10^5^ Geobacillus stearothermophilus (ATCC 7953;) (47). Between 6 and 12 BIs per cycle were placed throughout the room for each decontamination cycle. These were located behind or beneath equipment and surfaces, on the portable metal rack holding respirators, and either on or nested within the pairs of respirators to test aHP penetration (Fig. S3). After each cycle, each BI spore disc was transferred from its glassine/Tyvek envelope to tryptic soy broth (Spordex culture medium), incubated at 55°C, and analyzed after 7 days as an indicator of effective decontamination. There was one instance of a spore disc dropped during transfer, which resulted in a single positive BI from that cycle (cycle 5; see Table 4).

### Chemical indicators of H_2_O_2_.

Chemical indicator strips (Steris Steraffirm or CURIS system hydrogen peroxide test strips) were placed in various locations throughout the prep room (between 1 and 4 total per cycle) to indicate the presence of H_2_O_2_, supporting successful decontamination.

### Real-time hydrogen peroxide monitoring.

The portable ATI PortaSens II detector was used to measure H_2_O_2_ levels both within and outside the prep room during the decontamination process. Hydrogen peroxide concentrations were also measured at respirator surfaces and necessarily reduced to less than 1 ppm prior to handling and sealing for transportation to designated tissue culture rooms or for subsequent respirator fit-testing. During the charge and pulse periods of decontamination, real-time instantaneous sampling was conducted through a sealable wall port (designed for sterilizer tubing) into the prep room. The PortaSens was also used to monitor prep room concentrations at the start of the aeration phase and to verify that concentrations were reduced to less than 2 ppm for safe reentry to the room (without respiratory protection); these measurements were made at breathing zone height (BZH), or 5 feet above floor level. H_2_O_2_ concentrations were also monitored outside the prep room door seal (see Fig. S2) to check for any H_2_O_2_ leakage.

The U.S. Department of Labor/Occupational Safety and Health Administration (OSHA) and the American Conference of Governmental Industrial Hygienists (ACGIH) have established or adopted an 8-h time-weighted average (TWA) occupational permissible exposure limit (PEL) to hydrogen peroxide of 1 ppm (54, 55). Though researchers and fit-test subjects were not anticipated to experience this 8-h exposure level, a 2 ppm H_2_O_2_ concentration was used as a safety threshold for room entry, and <  ppm for removal of respirators, sealing respirators for transport, and reuse by study participants. See Text S1 (and Table S2) for additional metrics used to verify that research personnel were not adversely exposed to H_2_O_2._

10.1128/msphere.00303-22.1 TEXT S1 This text file contains methods and results on hydrogen peroxide diffusion sampling (by H_2_O_2_ vapor monitors [HPMs]), as well as additional methods on quantitative fit-testing metrics. Download Text S1, PDF file, 0.1 MB. Copyright © 2022 Derr et al. 2022 Derr et al. https://creativecommons.org/licenses/by/4.0/ This content is distributed under the terms of the Creative Commons Attribution 4.0 International license.

10.1128/msphere.00303-22.7 TABLE S2 Hydrogen peroxide diffusion sampler (HPM) data. The table lists the aHP cycle and location of HPMs, as well as the amount of H_2_O_2_ (ppm) observed and the duration of sampling. Download Table S2, PDF file, 0.1 MB. Copyright © 2022 Derr et al. 2022 Derr et al. https://creativecommons.org/licenses/by/4.0/ This content is distributed under the terms of the Creative Commons Attribution 4.0 International license.

### Respirator selection for the study.

The N95 respirator facepiece models examined in this study include a range of characteristics, including those identified as surgical N95 respirators (no exhalation valve to maintain sterile field), and nonsurgical N95 respirators with an exhalation valve (see Table 1 and Fig. S1). Respirator models were required to be successfully fitted by test subjects. Since fit test results varied widely by subject during early testing of Alpha Pro Tech respirators, this model was discontinued from further study. Fit-testing participants included experienced test subjects and/or administrators. Several respirator models were qualitatively fit-tested (QLFT) using OSHA fit test protocols (saccharine challenge) described in the OSHA respiratory protection standard (29 CFR 1910.134) (56). This included standard exercises to challenge respirator fit over an approximate 8-min, 30-s period. Additionally, quantitative fit-testing (QNFT) was conducted using OSHA protocol requirements with a TSI, Inc., PortaCount Pro+ model 8038 fit tester. This method employs condensation nucleus or particle counting technology (CNC or CPC) to measure aerosol concentration outside and inside the facepiece to determine a user fit factor (57).

### Sample size and acceptance criteria for fit-test reliability.

The study design for the QLFT endpoint was intended to rigorously evaluate user respirator to facepiece seal using the greatest-available pool of respirators (stockpiled model 3M 8511) while also providing representative feasibility data for the other respirator models (Table 1). The stockpiled 3M 8511 respirators were procured and collected by Penn State from 2006 to 2009. The use of these respirators beyond the manufacturer’s recommended shelf-life enabled the study to proceed without consuming respirators that were more urgently needed by frontline workers, and it was supported by CDC crisis-capacity scenarios in force at the time (40, 41). All other respirator models (Table 1) used in this study were within their expiration date. All respirators were new and unused at study initiation.

Acceptance criteria for 3M 8511 respirators required a minimum sample of 59 facepieces with no failures during QLFT in order to conclude with 95% confidence that at least 95% of 3M 8511 respirators maintain fit integrity after repeated use and decontamination. A total of 77 3M 8511 respirators were available for study purposes. A small number of respirators were allocated for QNFT and virology testing after QLFT, since these processes entailed respirator destruction (i.e., grommet insertion for QNFT or mask slicing for viral resuspension) and rendered them inaccessible for subsequent rounds of aHP.

### Respirator fit-testing.

Prior to decontamination, respirators were labeled with a unique identifier. After decontamination, a hash mark was placed on the lower elastic band of each respirator to identify the round(s) of decontamination completed (Fig. S2). Since the study was conducted during the COVID-19 pandemic, physical distancing and active clinical operating conditions limited the use of multiple test subjects. Therefore, two subjects were selected (one male, one female) to maximize various sizes and facial features for fit-testing. Respirators were subjected to QLFT on the first, fifth, and tenth rounds of decontamination. A small number of respirators per model were allocated for QNFT during each round of fit testing; due to installation of metal grommet/probe for QNFT, these could not be reused for subsequent aHP cycles or fit testing. These were repurposed for subsequent virus inactivation testing in order to conserve overall respirator use. See the Text S1 for additional details on QNFT metrics.

### NIOSH filtration efficiency testing.

To determine whether the N95 respirators used in this study experienced filtration medium breakdown as a result of sequential aHP disinfection cycles, several respirators were sent for independent laboratory analysis. These included eight 3M 8511 respirators subjected to cyclic aHP treatment and intermittent fit-testing and two additional unused, untreated 3M 8511 respirators. Respirator filtration efficiency testing was performed by ICS Laboratories, Inc., of Brunswick, Ohio. ICS is one of two firms in the United States authorized by the National Institute for Occupational Safety and Health (NIOSH) to perform respirator certification or recertification.

An abbreviated “short-cycle” filtration efficiency verification test was conducted using the NIOSH standard test procedure TEB-APR-STEP-0059 (58), which is summarized below. The standard test protocol includes initial respirator conditioning at 85 ± 5% relative humidity (%RH) and 38 ± 2°C for 25 h prior to testing. This conditioning was intended to reflect active moisture load created by respirator user. Following sealing of the respirator exhalation valve, and placement into the test instrument, full-load testing was performed. This testing included respirator challenge using 200 mg sodium chloride aerosol (mean count particle size distribution verified as 0.075 ± 0.020 μm, with the geometric standard deviation not exceeding 1.86). Sodium chloride aerosol was neutralized to a “Boltzman equilibrium state” (25 ± 5°C, 30 ± 10% relative humidity) and introduced at an airflow rate of 85 ± 4 L per minute, with periodic check and adjustment to maintain this flow rate. Instrumental analysis was conducted using a TSI automated filter tester model 8130A. Recorded data included flow rate, resistance, penetration, maximum penetration, and filtration efficiency.

The test protocol establishes the means for ensuring that the particulate filtering efficiency of N95 series filters used on nonpowered respirators submitted for approval, extension of approval, or examined during certified product audits, meets the minimum certification standards set forth in 42 CFR, Part 84, Subpart K, §84.181.

### Virus inoculation and titration.

The viruses used here included herpes simplex virus 1 (HSV-1) strain F, coxsackievirus B3 (CVB3), Pseudomonas phi6 bacteriophage (phi6), and SARS-CoV-2 isolate USA-WA1/2020. The key characteristics of these viruses are summarized in Table 2. All virus-inoculated materials were handled in accordance with the biosafety level (BSL) specified for that virus (see Table 2). Viruses were propagated in the same host cells as used for viral titration (see virus-specific sections below). In all aHP cycles, the respirators used for virus inactivation testing had been subjected to the preceding total number of aHP cycles (see Table 4 for details). The input inoculum for each virus was set to the maximum available in each viral stock preparation (Table 2).

For virus inactivation testing, respirator facepieces were inoculated with a controlled amount of one or more surrogate virus species (refer to Table 2 for complete list). Each virus was added in duplicate (or in quadruplicate during “post” cycles of testing; Table 3 and Table 4) droplets of 10 μL each on one or more surfaces of each respirator type. The zone of viral inoculation was marked at the corners, to allow excision of the inoculated area after aHP treatment or air-drying. Droplets were allowed to air dry or absorb fully onto each respirator inside a class II biosafety cabinet (BSC), before proceeding further. Selected samples of each virus-inoculated respirator were left under ambient conditions, without aHP decontamination, as a drying-only virus control. The remainder of each virus-inoculated facepiece was subjected to aHP decontamination as described above (i.e., decontaminated samples). For each round of viral testing, the duration of air-drying was matched to the duration of time needed for aHP treatment of parallel respirators (i.e., including transport, respirator staging in the prep room, aHP decontamination, aeration, and return to the viral testing location). After aHP treatment of respirators, each virus-inoculated area was cut out of the dried or decontaminated respirators using dissection scissors. Each excised virus spot encompassed all layers of the respirator, to capture any virus that had absorbed beyond the surface fabric.

### Herpes simplex virus 1 and coxsackievirus B3 quantification.

Excised respirator areas inoculated with HSV-1 or CVB3 were transferred into individual Eppendorf tubes and resuspended in a 250-μL volume of cell medium. Cell medium consisted of Dulbecco’s modified Eagle’s medium (DMEM) supplemented with 10% fetal bovine serum (FBS) and penicillin-streptomycin (Pen/Strep; 100 U/mL; Thermo Fisher Scientific). The number of infectious units, or PFU, for these viruses was determined by limiting dilution onto confluent Vero detector cell monolayers (Cercopithecus aethiops monkey kidney cells, ATCC CCL-81). Plaque formation was assessed at 72 h postinfection (hpi), after fixation and visualization of plaques using methylene blue staining. Duplicates of each virus-inoculated respirator piece were frozen for titration at a subsequent date, separate from the initial viral quantification. This allowed for experimental redundancy in terms of detector cell monolayers and reduced handling time for the large numbers of virus-respirator inoculation sites.

### Bacteriophage phi6 quantification.

Excised respirator areas inoculated with phi6 bacteriophage were transferred into individual Eppendorf tubes and resuspended in 250 μL of King’s medium B (KB). These were then quantified on lawns of Pseudomonas syringae pathovar phaseolicola strain 1448A (*Pph*) using a previously described soft agar overlay protocol (59). Briefly, 100 μL logarithmic culture of *Pph* (optical density at 600 nm [OD_600_], ~0.5) and 100 μL of the phage preparation were sequentially added to 3 mL of molten soft agar (0.7%) maintained at 55°C. The mixture was quickly poured on top of a KB agar plate and dried in the biosafety cabinet before transfer to a 28°C incubator. Alternatively, for enumeration of inoculum and dried samples, soft agar *Pph* lawns were prepared, and 10-μL dilutions of phage preparation were spotted. PFU were enumerated after 24 to 48 h of incubation at 28°C.

### SARS-coronavirus 2 quantification.

All experiments with SARS-CoV-2 were conducted in the Pell Laboratory under biosafety level 3 (BSL3) enhanced conditions using the USA-WA1/2020 isolate. SARS-CoV-2 was spotted onto masks in quadruplicate and treated with aHP or left under ambient BSL3 conditions for drying-only controls. Respirator areas inoculated with SARS-CoV-2 were then excised and resuspended in 250 μL of DMEM supplemented with sodium pyruvate, nonessential amino acids, antibiotics-antimycotics, and 2% FBS. After resuspension, viral titer was determined by a tissue culture infectious dose 50 (TCID_50_) assay in 96-well plates, using Vero E6 cells. Briefly, 20 μL of resuspended sample was added to 4 wells containing 180 μL of resuspension medium. The added samples were then serially diluted 10-fold down the plate, and the plates were incubated at 37°C for 96 h. At this time, cytopathic effect (CPE) was scored, and the titer was calculated using the method of Reed and Muench (60).
