# Supplementary figures and images for: Aerosolized Hydrogen Peroxide Decontamination of N95 Respirators, with Fit-Testing and Viral Inactivation, Demonstrates Feasibility for Reuse during the COVID-19 Pandemic
Source: mSphere. 2022 Aug 30;7(5):e00303-22. doi: 10.1128/msphere.00303-22 (PMC9599425; doi:10.1128/msphere.00303-22)

A. 1860 8511

Outside

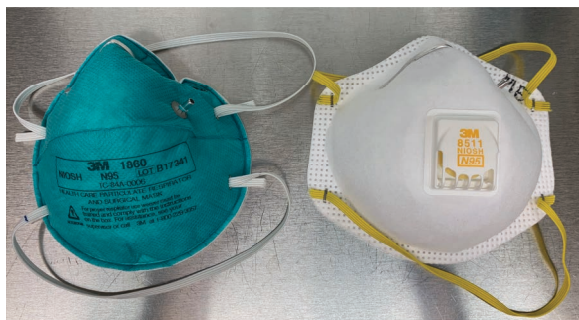

Inside

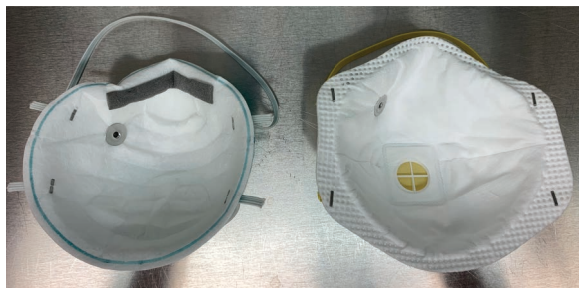

B. Virus-spotted examples

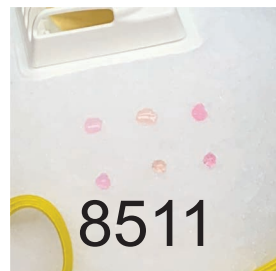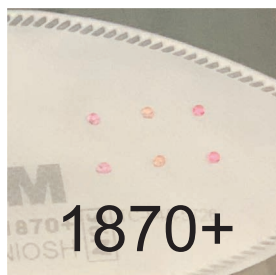

C. Outside

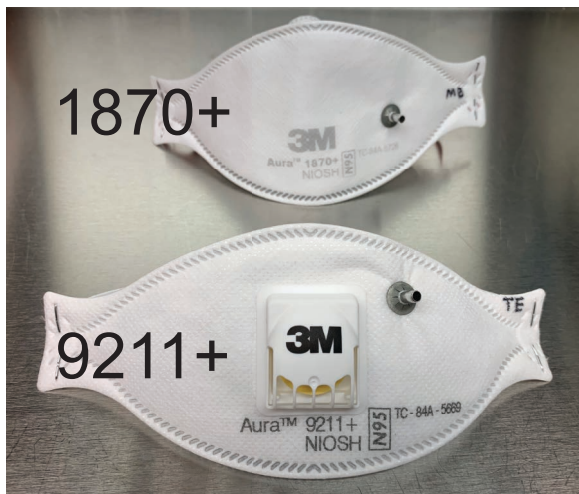

Inside

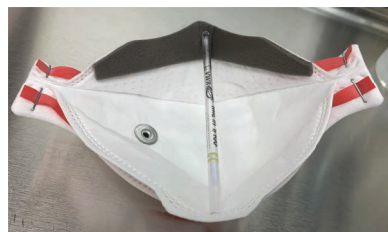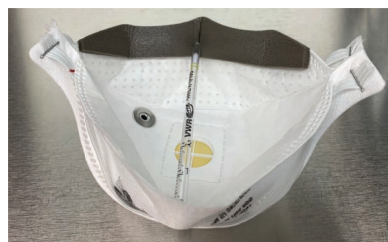

Supplement: FIG S1 [file msphere.00303-22-s0002.pdf]

A

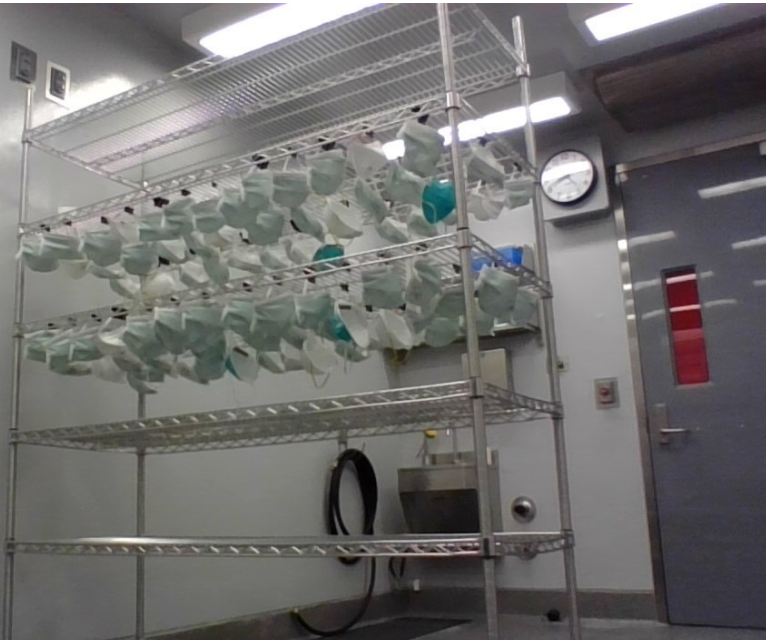

B

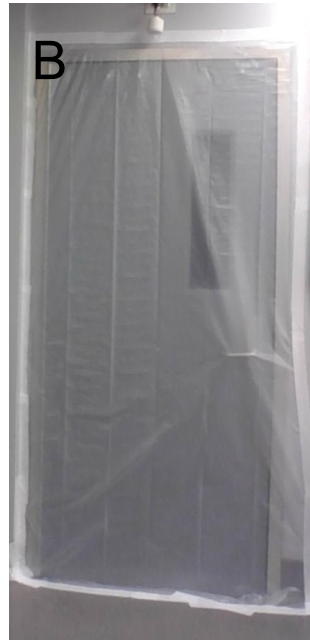

C

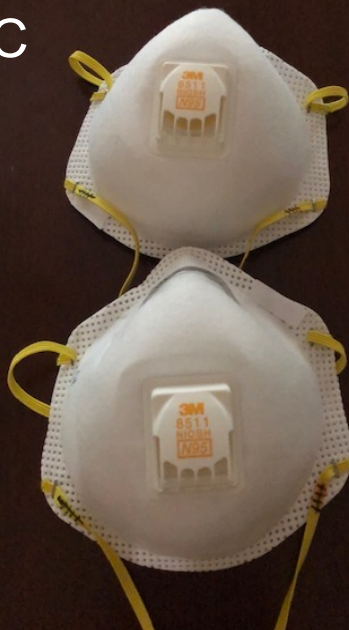

D

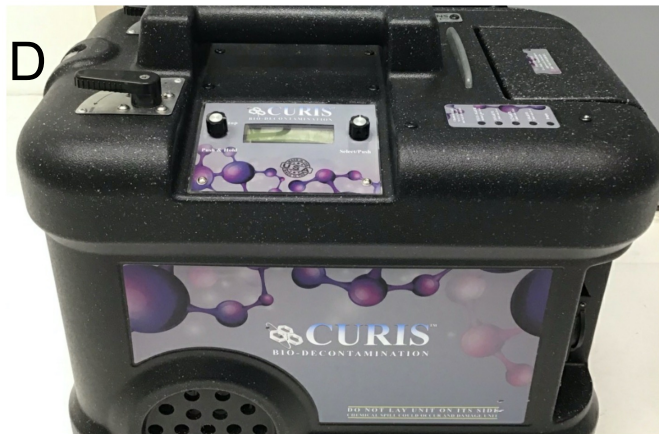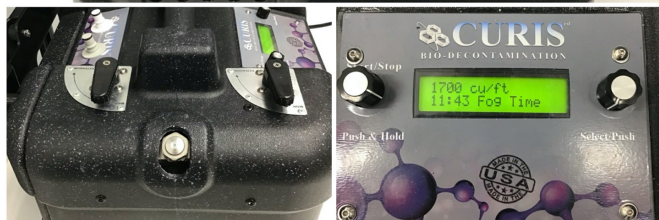

Supplement: FIG S2 [file msphere.00303-22-s0003.pdf]

**A**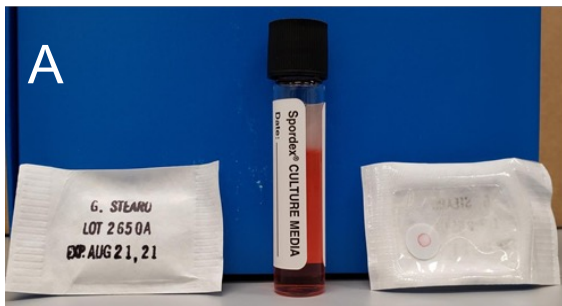**B**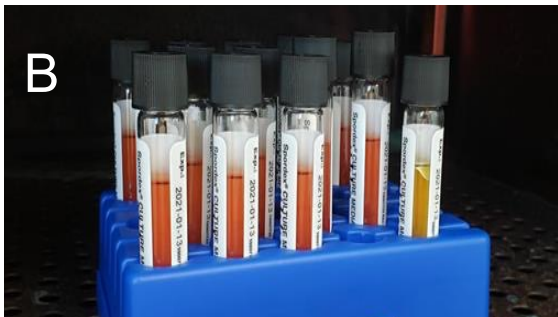**C**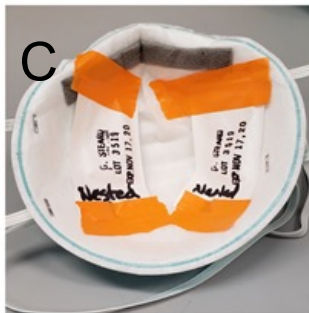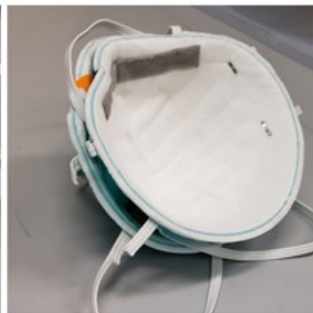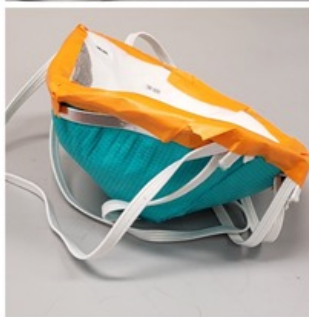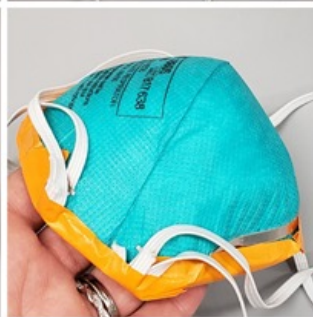

Supplement: FIG S3 [file msphere.00303-22-s0004.pdf]
