# Supplementary material for: Aerosolized Hydrogen Peroxide Decontamination of N95 Respirators, with Fit-Testing and Viral Inactivation, Demonstrates Feasibility for Reuse during the COVID-19 Pandemic
Source: mSphere. 2022 Aug 30;7(5):e00303-22. doi: 10.1128/msphere.00303-22 (PMC9599425; doi:10.1128/msphere.00303-22)
Supplement: FIG S4 [file msphere.00303-22-s0005.pdf]

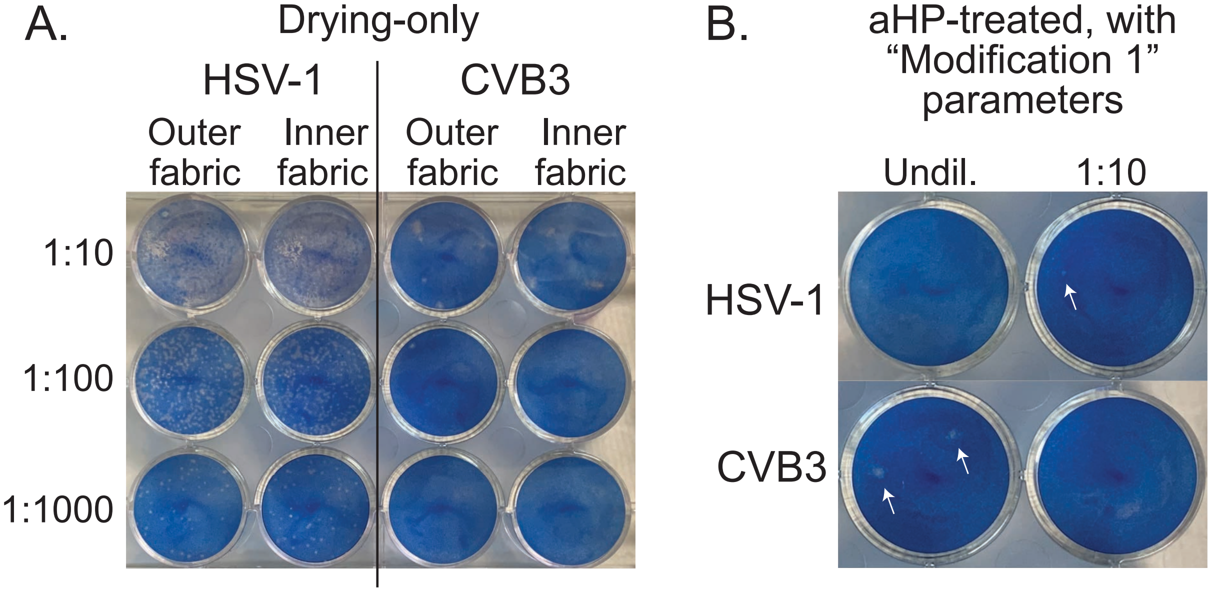
A.
Drying-only
HSV-1
CVB3
Outer fabric
Inner fabric
Outer fabric
Inner fabric
1:10
1:100
1:1000
B.
aHP-treated, with "Modification 1" parameters
Undil.
1:10
HSV-1
CVB3
