# Supplementary material for: Aerosolized Hydrogen Peroxide Decontamination of N95 Respirators, with Fit-Testing and Viral Inactivation, Demonstrates Feasibility for Reuse during the COVID-19 Pandemic
Source: mSphere. 2022 Aug 30;7(5):e00303-22. doi: 10.1128/msphere.00303-22 (PMC9599425; doi:10.1128/msphere.00303-22)
Supplement: TEXT S1 [file msphere.00303-22-s0001.pdf]

Supplementary Text

Methods for hydrogen peroxide diffusion sampling (by H_2O_2 vapor monitors)

Diffusion samplers, also known as hydrogen peroxide vapor monitors (HPMs) were utilized to monitor long-duration H_2O_2 leakage outside the Prep Room entry (outside containment or OC), on research personnel during decontamination activities (breathing zone samples, clipped to lapel-collar), and within sealed respirator transport containers (prior to fit-testing) to verify that H_2O_2 concentrations remained less than 1 ppm throughout transport and fit-testing. Once the decontamination process was standardized with specific room ventilation parameters, ongoing personnel monitoring was discontinued.

HPM sampler analysis was conducted by Advanced Chemical Sensors, Inc. (ACS) of Longwood, FL utilizing an ACS HP-10 hydrogen peroxide vapor monitor, via modified OSHA Method VI-6 (colorimetric analysis). Analysis was subcontracted and completed by laboratories participating in the American Industrial Hygiene Association (AIHA) Laboratory Accreditation Program. The AIHA is an ISO/IEC 17025 accrediting body (1, 2).

Additional methods on quantitative fit-testing metrics

Based on historical studies by the QNFT device and respirator manufacturers, N95 respirators have maximum breakthrough for particle sizes of 0.1 – 0.3 micron. Particles smaller or larger than this size range have increased filtration efficiency (greater than 95%). QNFT fit testing of N95 respirators therefore focuses on measuring this maximum penetration size range, to ensure that detectable leakage was due to facepiece seal leakage. OSHA has established a minimum QNFT passing fit factor of 100 for half-face respirators (including N95s or filtering-facepiece respirators) (3). The QNFT instrument manufacturer has established a maximum quantifiable fit factor of 200, as limited by measurement reliability and particle counting factors. QNFT fit factors equal to or exceeding 200 are therefore reported as 200(+). The reported QNFT data were utilized to verify successful fit reported by QLFT.

Results for hydrogen peroxide diffusion sampling (by H_2O_2 vapor monitors)

Diffusion samplers, also known as hydrogen peroxide vapor monitors (HPMs) were utilized to assess researcher exposure levels to H_2O_2, with reference to the OSHA permissible exposure limit (PEL) and ACGIH Threshold Limit Value (TLV®) of 1 ppm, as an eight-hour time-

weighted average (TWA). HPMs were also collected to monitor H_2O_2 build-up within respirator containers prior to fit-testing (for test subject safety), and to verify that H_2O_2 concentrations remained low in areas occupied by researchers (outside containment, OC) during the decontamination process (Table S2). All personal breathing zone HPM sampler results were reported below sample detection limits (< 0.08 ppm, <0.1 ppm) for researchers conducting aHP processes (up to 3 hours). All results for HPMs placed within sealed transport containers were reported below sample detection limits (< 0.03 ppm), indicating no residual H_2O_2 present through transport and respirator fit-testing. All results for HPMs collected as OC samples were reported below sample detection limits (< 0.08 ppm, cycle 1b) and < 0.2 ppm (cycle 11).

References

1. Advanced Chemical Sensors. AIHA-LAP ID No. 102047. Longwood, Florida.

2. SGS Galson Laboratories. AIHA-LAP ID No. 100324. E. Syracuse, New York 13057.

3. Occupational Safety and Health Administration (OSHA), United States Department of Labor. 2004. Fit Testing Procedures (Mandatory). 1910.134 App A. https://www.osha.gov/laws-regs/regulations/standardnumber/1910/1910.134AppA.
