# Supplementary material for: Aerosolized Hydrogen Peroxide Decontamination of N95 Respirators, with Fit-Testing and Viral Inactivation, Demonstrates Feasibility for Reuse during the COVID-19 Pandemic
Source: mSphere. 2022 Aug 30;7(5):e00303-22. doi: 10.1128/msphere.00303-22 (PMC9599425; doi:10.1128/msphere.00303-22)
Supplement: TABLE S2 [file msphere.00303-22-s0007.pdf]

Table S2: Hydrogen peroxide diffusion sampler (HPM) data

aHP Cycle ID	Sample Type	Location Notes*	H_2O_2 (ppm)**	Sampling Period (minutes)***
1a	Researcher A – personal breathing zone	Sampler at worker lapel; transit in all service areas	< 0.1	143
1b	Researcher B – personal breathing zone	Sampler at worker lapel; transit in all service areas	< 0.08	185
1b	area sample	Door Seal (OC), breathing zone height	< 0.08	183
1b	respirator container	3M 1860 (within brown paper bag)	< 0.03	461
1b	respirator container	3M 9211+ (bag)	< 0.03	518
1b	respirator container	Alpha Protech PFL (within brown paper bag)	< 0.03	497
1b	respirator container	Alpha Protech PFL (within brown paper bag)	< 0.03	510
11	area sample	Door Seal (OC), breathing zone height	< 0.2	110

*C (charge period), P (pulse period), D (dwell period), A (aeration phase)

** "< Value" indicates sample analysis was below limit of quantitation (LOQ) based on sampler diffusion rate and air volume collected. Time-weighted average (TWA) not calculated for results reported <LOQ. The OSHA PEL and ACGIH TLV® exposure standard for hydrogen peroxide is 1ppm eight-hour TWA.

***Sampling period varied by sampling objectives.
